# Supplementary material for: Multilevel analysis of factors associated with abortion among adolescents in Uganda insights from UDHS 2022 dataset
Source: PLOS Glob Public Health. 2026 Jan 12;6(1):e0005822. doi: 10.1371/journal.pgph.0005822 (PMC12795349; doi:10.1371/journal.pgph.0005822)
Supplement: Attachment — Submitted filename: Review comments.docx [file pgph.0005822.s001.docx]

**Review comments**

**Background**

- First paragraph- Authors state rightfully the problems of unintended pregnancies and its known associated factors (e.g. comprehensive sex education) but omit access to contraception as one of the key factors? This is a critical omission which risks a lack of balance to the arguments of safe abortion promotion to adolescents
- 2^nd^ paragraph- Subsequent fertility issues from termination of unwanted pregnancies must also be acknowledged
- Paragraph 5, 2^nd^ line: The word ‘be’ missing between the words ‘may’ and ‘influencing’

**Study population and data source:**

- Line 3: A word missing between ‘with’ and ‘Ministry of Health’
- Line 13: What does adolescents who ‘consented to ever had sex’ were included mean? Does consent in this context mean willing to participate in sexual activity? Does it therefore exclude adolescents who had non-consensual sex, e.g. rape and gender based related sexual activity? If not, consider rewording ‘consent’ to ‘ever having sex’ or ‘sexual debut’
- Please give examples of the dependent variables selected from literature in Uganda. You needn’t list them all but can give broad categories such as socio-economic, cultural, etc. to give readers an understanding of the variables included in the statistical analysis. Bear in mind that not all readers are familiar with the Ugandan context
- Last paragraph: Separate the combined words ‘whilethe’ in line two

**Multi-level model/ analysis**

- What does ‘increased region access of contraception’ mean in this context? What exactly is a region? Is it a district/ county? A City? A school catchment area? A clinic/ health facility catchment area? What would be the proximity to adolescent user to ascertain access? Etc.
- Since sexual debut seems really young for this population, what could be the associated factors? In particular, cultural practices (if any) like early marriage and gender based violence on children (particularly) those less than 15 should be explored. Does the DHS have gender based violence related variables/ data? Did authors consider this analysis?
  - The LR of 3.44 begs this question even more
- Any urban-rural differences in unsafe abortion prevalence’s since in the cluster sampling presented here, data was collected from both, making this comparative analysis possible. This could be helpful to unpack any unsafe abortion trends and link them to the differences in region, possibly to explain any access issues or other barriers to safe abortion where possible

**Assessing for variation in abortion at cluster level**

- Adolescents with no education had unsurprisingly the most odds of having an unsafe abortion. What was the regional LR. Was it similar across regions?

**Discussion**

- Paragraph 2: Very scanty. Authors should discuss what the characteristics of the variations in the regions of Uganda typically are. At the moment, authors have just declared that there are variations without getting into the arguments of it.
- Paragraph 3: Coercion into sex is stated by authors broadly. What is the Ugandan state of affairs as far as this is concerned? Any reported sexual violence against minors? Empirical studies? What about in contexts similar to Uganda e.g. neighboring East African countries? Etc.

**Limitations:**

- Authors should also acknowledge the non-consideration of gender based violence variables on a topic (unintended pregnancy and unsafe abortion practices of minors) where these often co-exist
- If available, authors should consider these in the analysis
