# Supplementary material for: Multilevel analysis of factors associated with abortion among adolescents in Uganda insights from UDHS 2022 dataset
Source: PLOS Glob Public Health. 2026 Jan 12;6(1):e0005822. doi: 10.1371/journal.pgph.0005822 (PMC12795349; doi:10.1371/journal.pgph.0005822)
Supplement: Attachment — Submitted filename: Response to Reviewers.docx [file pgph.0005822.s003.docx]

Dear Reviewers,

We thank you for the time and effort invested in reviewing our manuscript titled **Multilevel analysis of factors associated with abortion among adolescents in Uganda insights from UDHS 2022 Dataset**. We appreciate the insightful comments provided which have significantly strengthened the quality of our paper. Below, we have provided a point-by-point response to each comment raised by the reviewers. All revisions have been incorporated in the revised manuscript and updated accordingly as below.

**Reviewer Comments and Authors’ Responses**

**Background**

**1. Comment:**

- *First paragraph- Authors state rightfully the problems of unintended pregnancies and its known associated factors (e.g. comprehensive sex education) but omit access to contraception as one of the key factors? This is a critical omission which risks a lack of balance to the arguments of safe abortion promotion to adolescents*

**Response:**
Thank you for this important observation. We have revised the first paragraph to explicitly include access to contraception as a key determinant of unintended pregnancies among adolescents. This addition is on Page 3, paragraph 1 on Lines 4-5

**2. Comment:**

- *2^nd^ paragraph- Subsequent fertility issues from termination of unwanted pregnancies must also be acknowledged*

**Response:**
We agree with the reviewer. We have added a sentence acknowledging potential subsequent fertility complications associated with unsafe termination of pregnancies on Page 3, paragraph 2 Lines 7-9)

**3. Comment:**

- *Paragraph 5, 2^nd^ line: The word ‘be’ missing between the words ‘may’ and ‘influencing’*

**Response:**
The grammatical error was corrected. (Page 4, Line 2)

**Study Population and Data Source**

**4. Comment:**

*Line 3: A word missing between ‘with’ and ‘Ministry of Health’*

**Response:**
We have corrected this by inserting the word “**the**” (Page 4, Line 3, methodology section)

**5. Comment:**

- *Line 13: What does adolescents who ‘consented to ever had sex’ were included mean? Does consent in this context mean willing to participate in sexual activity? Does it therefore exclude adolescents who had non-consensual sex, e.g. rape and gender based related sexual activity? If not, consider rewording ‘consent’ to ‘ever having sex’ or ‘sexual debut’*

**Response:**
We thank the reviewer for this clarification and the wording was reshaped to “reported ever having sex”. (Page 5, Lines 13)

**6. Comment:**

- *Please give examples of the dependent variables selected from literature in Uganda. You needn’t list them all but can give broad categories such as socio-economic, cultural, etc. to give readers an understanding of the variables included in the statistical analysis. Bear in mind that not all readers are familiar with the Ugandan context*

**Response:**
We agree and have revised the manuscript to include broad categories of variables commonly used in similar studies in Uganda, such as socio-economic, demographic, cultural and health reproductive factors to help readers unfamiliar with the Ugandan context. (Page 5, Lines 5–8)

**7. Comment:**

- *Last paragraph: Separate the combined words ‘whilethe’ in line two*

**Response:**
Corrected as suggested. (Page 5, Paragraph 3, Line 2)

**Multilevel Model / Analysis**

**8. Comment:**

- *What does ‘increased region access of contraception’ mean in this context? What exactly is a region? Is it a district/ county? A City? A school catchment area? A clinic/ health facility catchment area? What would be the proximity to adolescent user to ascertain access? Etc.*

**Response:**
We appreciate this observation. We have revised the statement to clearly define “unmet need for contraception at the regional level is associated with higher likelihood…”. (Page 6, Lines 5–6)

**9. Comment:**

*Since sexual debut seems really young for this population, what could be the associated factors? In particular, cultural practices (if any) like early marriage and gender based violence on children (particularly) those less than 15 should be explored. Does the DHS have gender based violence related variables/ data? Did authors consider this analysis?*

**Response:**
Thank you for raising this important issue. We acknowledge that cultural practices such as early marriage and gender-based violence (GBV) may contribute to early sexual debut. However, the DHS 2022 dataset used for this analysis does **not** include GBV variables for adolescents. We have therefore added this as a limitation and clarified why these variables were not included in the analysis. (Page 13, Lines 6–9; Limitations section)

**10. Comment:**

- *Any urban-rural differences in unsafe abortion prevalence’s since in the cluster sampling presented here, data was collected from both, making this comparative analysis possible. This could be helpful to unpack any unsafe abortion trends and link them to the differences in region, possibly to explain any access issues or other barriers to safe abortion where possible*

**Response:**
We appreciate this suggestion and we have now included an explanation comparing unsafe abortion prevalence by urban and rural residence. (Page 6, last paragraph). For the multivariate analysis, variables with a bivariate association p < 0.1 were included (this is described in the methods section), which is why urban/rural is not presented in the multivariate analyses.

**Assessing Variation in Abortion at Cluster Level**

**11. Comment:**

- *Adolescents with no education had unsurprisingly the most odds of having an unsafe abortion. What was the regional LR. Was it similar across regions?*

**Response:**
We have updated and provided a response on. (Page 8, Lines 7–10, last paragraph)

**Discussion**

**12. Comment:**

- *Paragraph 2: Very scanty. Authors should discuss what the characteristics of the variations in the regions of Uganda typically are. At the moment, authors have just declared that there are variations without getting into the arguments of it.*

**Response:**
We have expanded this paragraph and provided a detailed discussion to elaborate on key socio-cultural, economic and service delivery differences across regions of Uganda that may explain the observed variations. (Page 11, Lines 3–11, paragraph 2 under discussion section)

**13. Comment:**

- *Paragraph 3: Coercion into sex is stated by authors broadly. What is the Ugandan state of affairs as far as this is concerned? Any reported sexual violence against minors? Empirical studies? What about in contexts similar to Uganda e.g. neighboring East African countries? Etc.*

**Response:**
We have revised the discussion to incorporate in depth evidence from Uganda and neighboring East African countries on sexual violence against minors including evidence from empirical studies (Page 12, Lines 4–13, paragraph 7)

**Limitations**

**14. Comment:**

- *Authors should also acknowledge the non-consideration of gender-based violence variables on a topic (unintended pregnancy and unsafe abortion practices of minors) where these often co-exist*

**Response:**
We agree and have added this important limitation. We have explained that the DHS dataset used did not include GBV variables applicable to the full adolescent population (most questions are only asked of women who have been married/partnered), which constrained our ability to incorporate them in an unbiased way. (Page 13, Lines 6–9)

**Conclusion**

We sincerely thank the reviewers and editor for the valuable comments provided. The manuscript has been thoroughly revised to address all the concerns raised. We trust that the revised version meets the expectations and standards of the journal.

Thank you

Yours Sincerely,

**Mungau Stephen**

Corresponding Author

On behalf of all co-authors
